# Ethyl 6-(4-bromo­phen­yl)-4-(4-fluoro­phen­yl)-2-oxocyclo­hex-3-ene-1-carboxyl­ate

**DOI:** 10.1107/S1600536812038202

**Published:** 2012-09-12

**Authors:** Rajni Kant, Vivek K. Gupta, Kamini Kapoor, M. Sapnakumari, B. Narayana, B. K. Sarojini

**Affiliations:** a X-ray Crystallography Laboratory, Post-Graduate Department of Physics & Electronics, University of Jammu, Jammu Tawi 180 006, India; b Department of Studies in Chemistry, Mangalore University, Mangalagangotri 574 199, India; c Department of Chemistry, P.A. College of Engineering, Nadupadavu, Mangalore 574153, India

## Abstract

There are two independent mol­ecules in the asymmetric unit of the title compound, C_21_H_18_BrFO_3_, in which the dihedral angles between the fluoro­phenyl and bromo­phenyl groups are 77.0 (1) and 85.8 (1)°. In one of the mol­ecules, two methine C—H groups of the cyclo­hexene ring are disordered over two sets of sites in a 0.53 (2):0.47 (2) ratio. In both mol­ecules, the atoms of the ethyl group were refined as disordered over two sets of sites with occupancies of 0.67 (2):0.33 (2) and 0.63 (4):0.37 (4). The cyclo­hexene rings have slightly distorted sofa conformations in both mol­ecules. In the crystal, C—H⋯O inter­actions link mol­ecules into chains along the *b* axis.

## Related literature

For background to the synthesis, see: Sreevidya *et al.* (2010 ▶); Padmavathi *et al.* (2000 ▶); Senguttuvan & Nagarajan (2010 ▶); Butcher *et al.* (2011 ▶). For related structures, see: Dutkiewicz *et al.* (2011*a* ▶,*b* ▶,*c* ▶); Fun *et al.* (2010 ▶); Harrison *et al.* (2010 ▶). For ring conformations, see: Duax & Norton (1975 ▶).
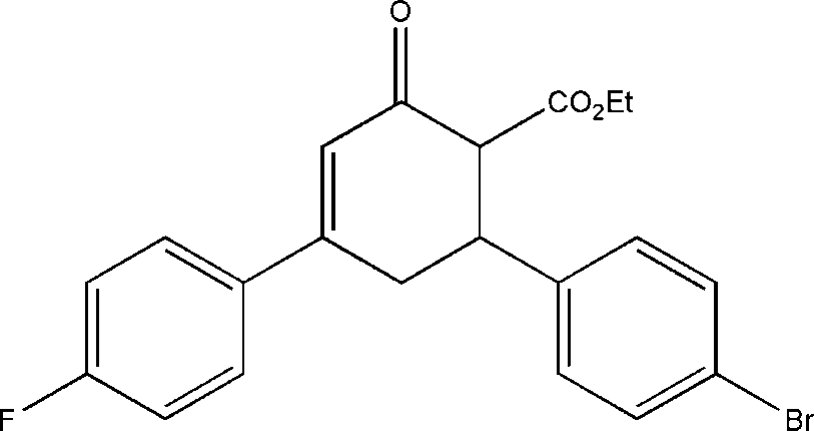


## Experimental

### 

#### Crystal data

C_21_H_18_BrFO_3_
*M* *_r_* = 417.26
Triclinic, 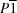

*a* = 11.8886 (5) Å
*b* = 13.3481 (5) Å
*c* = 13.4128 (5) Å
α = 77.214 (3)°
β = 66.757 (4)°
γ = 87.856 (3)°
*V* = 1904.27 (13) Å^3^
*Z* = 4
Mo *K*α radiation
μ = 2.19 mm^−1^
*T* = 293 K
0.3 × 0.2 × 0.2 mm

#### Data collection

Oxford Diffraction Xcalibur Sapphire3 diffractometer
Absorption correction: multi-scan (*CrysAlis PRO*; Oxford Diffraction, 2010 ▶) *T*_min_ = 0.816, *T*_max_ = 1.000
27878 measured reflections
7484 independent reflections
4086 reflections with *I* > 2σ(*I*)
*R*_int_ = 0.049

#### Refinement

*R*[*F* ^2^ > 2σ(*F* ^2^)] = 0.054
*wR*(*F* ^2^) = 0.139
*S* = 1.02
7484 reflections
526 parameters
8 restraints
H-atom parameters constrained
Δρ_max_ = 0.49 e Å^−3^
Δρ_min_ = −0.52 e Å^−3^

### 

Data collection: *CrysAlis PRO* (Oxford Diffraction, 2010 ▶); cell refinement: *CrysAlis PRO*; data reduction: *CrysAlis PRO*; program(s) used to solve structure: *SHELXS97* (Sheldrick, 2008 ▶); program(s) used to refine structure: *SHELXL97* (Sheldrick, 2008 ▶); molecular graphics: *ORTEP-3* (Farrugia, 1997 ▶); software used to prepare material for publication: *PLATON* (Spek, 2009 ▶).

## Supplementary Material

Crystal structure: contains datablock(s) I, global. DOI: 10.1107/S1600536812038202/gk2518sup1.cif

Structure factors: contains datablock(s) I. DOI: 10.1107/S1600536812038202/gk2518Isup2.hkl

Supplementary material file. DOI: 10.1107/S1600536812038202/gk2518Isup3.cml

Additional supplementary materials:  crystallographic information; 3D view; checkCIF report

## Figures and Tables

**Table 1:** Hydrogen-bond geometry (Å, °)

*D*—H⋯*A*	*D*—H	H⋯*A*	*D*⋯*A*	*D*—H⋯*A*
C5*A*—H5*A*1⋯O1*B* ^i^	0.97	2.58	3.388 (5)	141
C14*A*—H14*A*⋯O1*B* ^i^	0.93	2.58	3.445 (6)	154
C5*B*—H5*B*1⋯O1*A* ^ii^	0.97	2.55	3.351 (4)	140
C5*B*—H5*B*2⋯O2*A* ^iii^	0.97	2.59	3.457 (5)	149

Symmetry codes: (i) 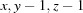; (ii) 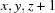; (iii) 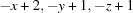.

**Fig. 1.**
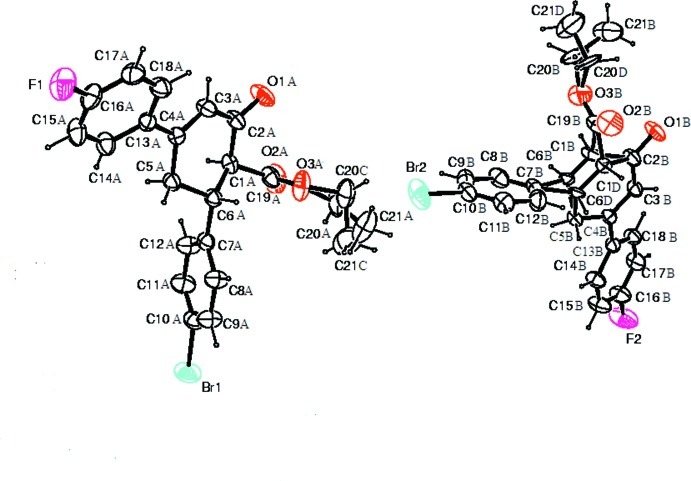
The molecular structure of (I) with ellipsoids drawn at the 40% probability level. H atoms are shown as small spheres of arbitrary radii.

**Fig. 2.**
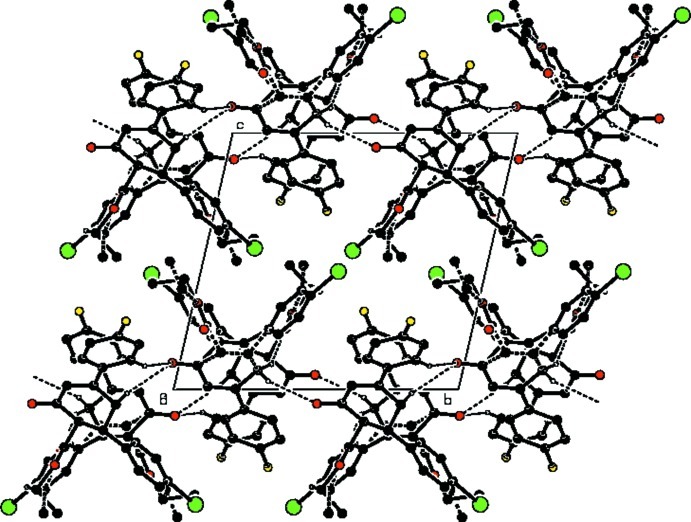
The packing arrangement of molecules viewed along the a axis. The broken lines show intermolecular C—H···O interactions.

## supplementary crystallographic information

### Comment

Michael addition of ethyl acetoacetate to chalcones yields 4,6-diaryl-2-oxo -cyclohex-3-ene-1-carboxylate derivatives (Sreevidya *et al.*, 2010), which could be used as efficient synthons for building spiro compounds or as intermediates in the synthesis of isoxazoles, pyrazoles and quinazolines (Padmavathi *et al.*, 2000; Senguttuvan & Nagarajan,2010; Butcher *et al.*, 2011). The crystal structure of some cyclohexenone derivatives, *viz*., methyl 4,6-bis(4-fluorophenyl)-2-oxo cyclohex-3-ene-1-carboxylate (Fun *et al.*, 2010), (1RS,6SR)-ethyl 4-(4-chlorophenyl)-6-(4-fluorophenyl)-2-oxocyclohex-3-ene-1-carboxylate toluene hemisolvate (Dutkiewicz *et al.*, 2011*a*), (1RS,6SR)-ethyl 4,6-bis(4-fluorophenyl)-2-oxocyclohex-3]-ene-1-carboxylate (Dutkiewicz *et al.*, 2011*b*), (1RS,6SR)-ethyl 4-(2,4-dichlorophenyl)-6-(4-fluorophenyl) -2-oxocyclohex-3-ene-1-carboxylate (Dutkiewicz *et al.*,2011*c*) and ethyl 4-(2,4-dichlorophenyl)-6-(6-methoxy-2-naphthyl)-2-oxocyclohex-3-ene-1- carboxylate(Harrison *et al.*, 2010) have been reported. In view of the importance of cyclohexenone derivatives, the title compound (I) was prepared and its crystal structure is reported.

In (I) (Fig. 1), all bond lengths and angles are normal and correspond to those to those observed in the related structures (Dutkiewicz *et al.*, 2011*a*,*b*,*c*; Fun *et al.*, 2010; Harrison *et al.*, 2010). The cyclohexene rings have slightly distorted sofa conformations in both the molecules [asymmetry parameters: ΔCs(C3A—C6A) = 4.11 for molecule A; ΔCs(C2B—C5B) = 4.58 /4.65 for molecule B (Duax & Norton, 1975)]. The fluorophenyl and bromophenyl rings are inclined to each other forming dihedral angles of 77.0 (1)° in molecule A and 85.8 (1)° in molecule B. In molecule B, C1 and C6 are disordered over two sites with a 0.53 (2):0.47 (2) ratio. The atoms of the ethyl group were refined as disordered over two sets of sites with occupancies of 0.67 (2)/0.33 (2) and 0.63 (4)/0.37 (4). In the crystal structure C—H···O hydrogen bonds create chains of molecules along the *y* direction (Table 1, Fig. 2).

### Experimental

(2E)-3-(4-Bromophenyl)-1-(4-fluorophenyl)prop-2-en-1-one (3.05 g, 0.01 mol) and ethyl acetoacetate (1.30 g, 0.01 mol) were refluxed for 8 h in 30 ml absolute alcohol in presence of 10% NaOH. The reaction mixture was cooled to room temperature and the precipitate obtained was filtered. Single crystals were grown by slow evaporation from absolute alcohol (m.p.= 403 K).

### Refinement

All H atoms were positioned geometrically and were treated as riding on their parent C atoms, with C—H distances of 0.93–0.98 Å and with *U*_iso_(H) = 1.2*U*_eq_(C) or 1.5*U*_eq_(methyl C). Iin the refinement process restraints were imposed on C-C [1.52 (1) Å] and C-O [1.42 (1) Å] distances of the disordered molecular fragments.

### Crystal data

**Table:**

C_21_H_18_BrFO_3_	*Z* = 4
*M**_r_* = 417.26	*F*(000) = 848
Triclinic, *P*1	*D*_x_ = 1.455 Mg m^−^^3^
Hall symbol: -P 1	Mo *K*α radiation, λ = 0.71073 Å
*a* = 11.8886 (5) Å	Cell parameters from 7575 reflections
*b* = 13.3481 (5) Å	θ = 3.4–29.0°
*c* = 13.4128 (5) Å	µ = 2.19 mm^−^^1^
α = 77.214 (3)°	*T* = 293 K
β = 66.757 (4)°	Block, colourless
γ = 87.856 (3)°	0.3 × 0.2 × 0.2 mm
*V* = 1904.27 (13) Å^3^	

### Data collection

**Table:**

Oxford Diffraction Xcalibur Sapphire3 diffractometer	7484 independent reflections
Radiation source: fine-focus sealed tube	4086 reflections with *I* > 2σ(*I*)
Graphite monochromator	*R*_int_ = 0.049
Detector resolution: 16.1049 pixels mm^-1^	θ_max_ = 26.0°, θ_min_ = 3.4°
ω scans	*h* = −14→14
Absorption correction: multi-scan (*CrysAlis PRO*; Oxford Diffraction, 2010)	*k* = −16→16
*T*_min_ = 0.816, *T*_max_ = 1.000	*l* = −16→16
27878 measured reflections	

### Refinement

**Table:**

Refinement on *F*^2^	Primary atom site location: structure-invariant direct methods
Least-squares matrix: full	Secondary atom site location: difference Fourier map
*R*[*F*^2^ > 2σ(*F*^2^)] = 0.054	Hydrogen site location: inferred from neighbouring sites
*wR*(*F*^2^) = 0.139	H-atom parameters constrained
*S* = 1.02	*w* = 1/[σ^2^(*F*_o_^2^) + (0.0428*P*)^2^ + 0.8556*P*] where *P* = (*F*_o_^2^ + 2*F*_c_^2^)/3
7484 reflections	(Δ/σ)_max_ = 0.002
526 parameters	Δρ_max_ = 0.49 e Å^−^^3^
8 restraints	Δρ_min_ = −0.52 e Å^−^^3^

### Special details

**Table:**

Experimental. *CrysAlis PRO*, Oxford Diffraction Ltd., Version 1.171.34.40 (release 27–08-2010 CrysAlis171. NET) (compiled Aug 27 2010,11:50:40) Empirical absorption correction using spherical harmonics, implemented in SCALE3 ABSPACK scaling algorithm.
Geometry. All e.s.d.'s (except the e.s.d. in the dihedral angle between two l.s. planes) are estimated using the full covariance matrix. The cell e.s.d.'s are taken into account individually in the estimation of e.s.d.'s in distances, angles and torsion angles; correlations between e.s.d.'s in cell parameters are only used when they are defined by crystal symmetry. An approximate (isotropic) treatment of cell e.s.d.'s is used for estimating e.s.d.'s involving l.s. planes.
Refinement. Refinement of *F*^2^ against ALL reflections. The weighted *R*-factor *wR* and goodness of fit *S* are based on *F*^2^, conventional *R*-factors *R* are based on *F*, with *F* set to zero for negative *F*^2^. The threshold expression of *F*^2^ > σ(*F*^2^) is used only for calculating *R*-factors(gt) *etc*. and is not relevant to the choice of reflections for refinement. *R*-factors based on *F*^2^ are statistically about twice as large as those based on *F*, and *R*- factors based on ALL data will be even larger.

### Fractional atomic coordinates and isotropic or equivalent isotropic displacement parameters (Å^2^)

**Table:**

	*x*	*y*	*z*	*U*_iso_*/*U*_eq_	Occ. (<1)
Br1	0.77287 (6)	−0.17028 (4)	0.44941 (5)	0.1056 (2)	
Br2	0.75908 (7)	0.51689 (5)	0.54046 (6)	0.1305 (3)	
F1	0.3550 (3)	0.2225 (2)	−0.2653 (2)	0.1048 (10)	
F2	1.0871 (3)	0.6057 (2)	1.3044 (3)	0.1287 (12)	
O1A	0.7343 (3)	0.4848 (2)	0.0562 (3)	0.0910 (10)	
O2A	0.8428 (3)	0.3445 (3)	0.2372 (3)	0.1018 (12)	
O3A	0.6423 (3)	0.3555 (3)	0.2950 (3)	0.0949 (11)	
C1A	0.7418 (3)	0.3066 (3)	0.1261 (3)	0.0520 (9)	
H1A	0.8232	0.2872	0.0806	0.062*	
C2A	0.7087 (4)	0.3978 (3)	0.0551 (3)	0.0598 (10)	
C3A	0.6499 (3)	0.3773 (3)	−0.0146 (3)	0.0577 (10)	
H3A	0.6352	0.4329	−0.0626	0.069*	
C4A	0.6151 (3)	0.2825 (3)	−0.0142 (3)	0.0464 (9)	
C5A	0.6395 (3)	0.1900 (3)	0.0593 (3)	0.0501 (9)	
H5A1	0.7143	0.1612	0.0161	0.060*	
H5A2	0.5730	0.1384	0.0844	0.060*	
C6A	0.6521 (3)	0.2134 (3)	0.1607 (3)	0.0496 (9)	
H6A	0.5716	0.2326	0.2081	0.060*	
C7A	0.6846 (4)	0.1189 (3)	0.2291 (3)	0.0507 (9)	
C8A	0.5945 (4)	0.0566 (3)	0.3168 (3)	0.0676 (12)	
H8A	0.5133	0.0732	0.3343	0.081*	
C9A	0.6201 (5)	−0.0302 (4)	0.3803 (4)	0.0795 (14)	
H9A	0.5564	−0.0724	0.4380	0.095*	
C10A	0.7393 (5)	−0.0543 (3)	0.3583 (4)	0.0652 (11)	
C11A	0.8305 (4)	0.0051 (3)	0.2706 (4)	0.0759 (13)	
H11A	0.9117	−0.0115	0.2534	0.091*	
C12A	0.8028 (4)	0.0903 (3)	0.2068 (4)	0.0743 (13)	
H12A	0.8665	0.1299	0.1464	0.089*	
C13A	0.5498 (3)	0.2674 (3)	−0.0844 (3)	0.0497 (9)	
C14A	0.5490 (4)	0.1739 (3)	−0.1135 (3)	0.0630 (11)	
H14A	0.5926	0.1208	−0.0908	0.076*	
C15A	0.4850 (4)	0.1583 (4)	−0.1752 (4)	0.0752 (13)	
H15A	0.4853	0.0956	−0.1947	0.090*	
C16A	0.4208 (4)	0.2374 (4)	−0.2072 (3)	0.0703 (12)	
C17A	0.4188 (4)	0.3300 (4)	−0.1807 (3)	0.0704 (12)	
H17A	0.3752	0.3827	−0.2039	0.084*	
C18A	0.4830 (4)	0.3439 (3)	−0.1188 (3)	0.0636 (11)	
H18A	0.4815	0.4068	−0.0994	0.076*	
C19A	0.7507 (5)	0.3369 (3)	0.2249 (4)	0.0639 (11)	
O1B	0.7894 (3)	1.0258 (2)	0.8969 (3)	0.0813 (9)	
O2B	0.6448 (3)	0.9414 (3)	0.7664 (3)	0.1059 (12)	
O3B	0.8394 (3)	0.9849 (3)	0.6646 (3)	0.0984 (11)	
C2B	0.8113 (4)	0.9352 (3)	0.9076 (3)	0.0615 (11)	
C3B	0.8562 (3)	0.8817 (3)	0.9891 (3)	0.0524 (9)	
H3B	0.8648	0.9176	1.0380	0.063*	
C4B	0.8864 (3)	0.7835 (3)	0.9984 (3)	0.0448 (9)	
C5B	0.8690 (3)	0.7201 (3)	0.9254 (3)	0.0483 (9)	
H5B1	0.7959	0.6749	0.9676	0.058*	
H5B2	0.9385	0.6776	0.9020	0.058*	
C7B	0.8113 (5)	0.7136 (3)	0.7658 (4)	0.0650 (12)	
C8B	0.9083 (5)	0.6896 (3)	0.6797 (4)	0.0730 (13)	
H8B	0.9867	0.7139	0.6652	0.088*	
C9B	0.8935 (4)	0.6307 (3)	0.6137 (4)	0.0737 (13)	
H9B	0.9614	0.6145	0.5560	0.088*	
C10B	0.7785 (5)	0.5958 (3)	0.6333 (4)	0.0657 (12)	
C11B	0.6797 (4)	0.6188 (4)	0.7187 (4)	0.0741 (12)	
H11B	0.6012	0.5952	0.7322	0.089*	
C12B	0.6965 (5)	0.6773 (4)	0.7847 (4)	0.0791 (13)	
H12B	0.6288	0.6924	0.8434	0.095*	
C13B	0.9384 (3)	0.7348 (3)	1.0797 (3)	0.0488 (9)	
C14B	0.9309 (4)	0.6287 (3)	1.1197 (3)	0.0649 (11)	
H14B	0.8917	0.5866	1.0951	0.078*	
C15B	0.9806 (4)	0.5851 (3)	1.1950 (4)	0.0834 (14)	
H15B	0.9752	0.5142	1.2214	0.100*	
C16B	1.0380 (4)	0.6482 (4)	1.2301 (4)	0.0754 (13)	
C17B	1.0500 (4)	0.7517 (3)	1.1916 (4)	0.0723 (12)	
H17B	1.0913	0.7926	1.2155	0.087*	
C18B	1.0003 (4)	0.7949 (3)	1.1171 (3)	0.0599 (10)	
H18B	1.0079	0.8659	1.0907	0.072*	
C19B	0.7485 (5)	0.9349 (3)	0.7526 (4)	0.0682 (12)	
C1B	0.825 (2)	0.8778 (8)	0.8156 (15)	0.065 (4)	0.53 (2)
H1B	0.9043	0.9110	0.7622	0.078*	0.53 (2)
C6B	0.8566 (13)	0.7901 (9)	0.8195 (11)	0.043 (2)	0.53 (2)
H6B	0.9431	0.8023	0.7682	0.051*	0.53 (2)
C1D	0.7572 (13)	0.8634 (10)	0.8570 (11)	0.041 (3)	0.47 (2)
H1D	0.6712	0.8524	0.9094	0.049*	0.47 (2)
C6D	0.795 (2)	0.7666 (9)	0.8617 (14)	0.051 (4)	0.47 (2)
H6D	0.7165	0.7314	0.9145	0.062*	0.47 (2)
C20B	0.825 (2)	1.0523 (11)	0.5699 (10)	0.139 (6)	0.670 (17)
H20C	0.7481	1.0363	0.5669	0.167*	0.670 (17)
H20D	0.8917	1.0455	0.5010	0.167*	0.670 (17)
C21B	0.8291 (12)	1.1592 (8)	0.5877 (12)	0.133 (6)	0.670 (17)
H21A	0.8139	1.2075	0.5303	0.199*	0.670 (17)
H21B	0.9083	1.1755	0.5852	0.199*	0.670 (17)
H21C	0.7673	1.1629	0.6591	0.199*	0.670 (17)
C20D	0.7758 (19)	1.053 (2)	0.608 (2)	0.110 (10)	0.330 (17)
H20E	0.7269	1.0972	0.6550	0.132*	0.330 (17)
H20F	0.7213	1.0127	0.5918	0.132*	0.330 (17)
C21D	0.868 (2)	1.117 (3)	0.501 (2)	0.160 (12)	0.330 (17)
H21D	0.8261	1.1610	0.4617	0.240*	0.330 (17)
H21E	0.9172	1.0723	0.4548	0.240*	0.330 (17)
H21F	0.9204	1.1579	0.5173	0.240*	0.330 (17)
C20A	0.659 (3)	0.371 (4)	0.3923 (19)	0.098 (9)	0.37 (4)
H20A	0.6931	0.4398	0.3788	0.117*	0.37 (4)
H20B	0.7130	0.3215	0.4113	0.117*	0.37 (4)
C21A	0.533 (3)	0.355 (5)	0.484 (3)	0.126 (14)	0.37 (4)
H21G	0.5354	0.3748	0.5481	0.190*	0.37 (4)
H21H	0.5050	0.2844	0.5038	0.190*	0.37 (4)
H21I	0.4772	0.3974	0.4595	0.190*	0.37 (4)
C20C	0.624 (2)	0.4003 (15)	0.3906 (11)	0.099 (6)	0.63 (4)
H20G	0.5584	0.4478	0.4021	0.119*	0.63 (4)
H20H	0.6982	0.4356	0.3814	0.119*	0.63 (4)
C21C	0.589 (4)	0.3070 (16)	0.484 (2)	0.150 (9)	0.63 (4)
H21J	0.5817	0.3264	0.5513	0.225*	0.63 (4)
H21K	0.6517	0.2583	0.4668	0.225*	0.63 (4)
H21L	0.5125	0.2765	0.4946	0.225*	0.63 (4)

### Atomic displacement parameters (Å^2^)

**Table:**

	*U*^11^	*U*^22^	*U*^33^	*U*^12^	*U*^13^	*U*^23^
Br1	0.1496 (6)	0.0713 (4)	0.1123 (5)	0.0181 (3)	−0.0823 (4)	0.0029 (3)
Br2	0.1991 (7)	0.1141 (5)	0.1657 (6)	0.0466 (5)	−0.1374 (6)	−0.0866 (5)
F1	0.112 (2)	0.141 (3)	0.105 (2)	0.0235 (19)	−0.0783 (18)	−0.0513 (19)
F2	0.194 (3)	0.093 (2)	0.156 (3)	0.007 (2)	−0.145 (3)	0.001 (2)
O1A	0.130 (3)	0.0419 (17)	0.130 (3)	−0.0011 (18)	−0.080 (2)	−0.0210 (18)
O2A	0.089 (2)	0.124 (3)	0.137 (3)	0.021 (2)	−0.074 (2)	−0.064 (2)
O3A	0.081 (2)	0.133 (3)	0.090 (2)	0.005 (2)	−0.0300 (19)	−0.070 (2)
C1A	0.054 (2)	0.047 (2)	0.063 (2)	0.0052 (18)	−0.0262 (19)	−0.0231 (19)
C2A	0.070 (3)	0.039 (2)	0.080 (3)	0.005 (2)	−0.036 (2)	−0.019 (2)
C3A	0.071 (3)	0.039 (2)	0.071 (3)	0.0051 (19)	−0.039 (2)	−0.0081 (19)
C4A	0.048 (2)	0.045 (2)	0.046 (2)	0.0056 (17)	−0.0174 (17)	−0.0124 (17)
C5A	0.061 (2)	0.043 (2)	0.056 (2)	0.0043 (18)	−0.0306 (19)	−0.0149 (18)
C6A	0.057 (2)	0.045 (2)	0.055 (2)	0.0024 (18)	−0.0284 (19)	−0.0141 (18)
C7A	0.061 (3)	0.047 (2)	0.056 (2)	0.0033 (19)	−0.034 (2)	−0.0153 (19)
C8A	0.057 (3)	0.059 (3)	0.074 (3)	−0.001 (2)	−0.022 (2)	0.002 (2)
C9A	0.085 (3)	0.065 (3)	0.074 (3)	−0.005 (3)	−0.027 (3)	0.008 (2)
C10A	0.088 (3)	0.049 (2)	0.073 (3)	0.006 (2)	−0.052 (3)	−0.008 (2)
C11A	0.066 (3)	0.068 (3)	0.095 (3)	0.008 (2)	−0.042 (3)	−0.002 (3)
C12A	0.064 (3)	0.065 (3)	0.079 (3)	0.000 (2)	−0.026 (2)	0.008 (2)
C13A	0.056 (2)	0.052 (2)	0.046 (2)	0.0076 (19)	−0.0226 (19)	−0.0148 (18)
C14A	0.070 (3)	0.065 (3)	0.073 (3)	0.020 (2)	−0.041 (2)	−0.030 (2)
C15A	0.084 (3)	0.084 (3)	0.086 (3)	0.019 (3)	−0.049 (3)	−0.048 (3)
C16A	0.069 (3)	0.100 (4)	0.057 (3)	0.011 (3)	−0.035 (2)	−0.030 (3)
C17A	0.080 (3)	0.078 (3)	0.064 (3)	0.021 (3)	−0.043 (2)	−0.012 (2)
C18A	0.073 (3)	0.056 (3)	0.070 (3)	0.009 (2)	−0.038 (2)	−0.012 (2)
C19A	0.072 (3)	0.054 (3)	0.084 (3)	0.008 (2)	−0.045 (3)	−0.026 (2)
O1B	0.122 (3)	0.0407 (17)	0.115 (2)	0.0241 (16)	−0.077 (2)	−0.0292 (17)
O2B	0.089 (3)	0.113 (3)	0.129 (3)	0.000 (2)	−0.058 (2)	−0.027 (2)
O3B	0.100 (3)	0.097 (3)	0.095 (3)	−0.004 (2)	−0.040 (2)	−0.012 (2)
C2B	0.081 (3)	0.040 (2)	0.077 (3)	0.007 (2)	−0.043 (2)	−0.018 (2)
C3B	0.062 (2)	0.044 (2)	0.062 (2)	0.0060 (18)	−0.031 (2)	−0.0206 (19)
C4B	0.043 (2)	0.040 (2)	0.054 (2)	0.0004 (16)	−0.0208 (17)	−0.0122 (17)
C5B	0.054 (2)	0.038 (2)	0.062 (2)	0.0081 (17)	−0.0297 (19)	−0.0156 (18)
C7B	0.106 (4)	0.043 (2)	0.076 (3)	0.023 (2)	−0.063 (3)	−0.024 (2)
C8B	0.083 (3)	0.059 (3)	0.098 (4)	0.006 (2)	−0.055 (3)	−0.022 (3)
C9B	0.083 (3)	0.076 (3)	0.075 (3)	0.020 (3)	−0.038 (3)	−0.033 (3)
C10B	0.099 (4)	0.049 (2)	0.081 (3)	0.021 (2)	−0.065 (3)	−0.025 (2)
C11B	0.078 (3)	0.071 (3)	0.085 (3)	0.005 (3)	−0.043 (3)	−0.020 (3)
C12B	0.097 (4)	0.083 (3)	0.069 (3)	0.021 (3)	−0.037 (3)	−0.034 (3)
C13B	0.054 (2)	0.046 (2)	0.051 (2)	0.0056 (18)	−0.0234 (19)	−0.0145 (18)
C14B	0.079 (3)	0.048 (2)	0.084 (3)	−0.001 (2)	−0.052 (2)	−0.010 (2)
C15B	0.113 (4)	0.052 (3)	0.104 (4)	−0.002 (3)	−0.073 (3)	0.001 (3)
C16B	0.099 (3)	0.063 (3)	0.087 (3)	0.004 (3)	−0.066 (3)	−0.005 (3)
C17B	0.096 (3)	0.061 (3)	0.083 (3)	0.001 (2)	−0.057 (3)	−0.019 (2)
C18B	0.079 (3)	0.047 (2)	0.066 (3)	0.004 (2)	−0.041 (2)	−0.013 (2)
C19B	0.094 (4)	0.047 (3)	0.088 (4)	0.012 (3)	−0.062 (3)	−0.017 (2)
C1B	0.099 (12)	0.029 (5)	0.085 (9)	−0.008 (6)	−0.060 (9)	−0.002 (5)
C6B	0.053 (6)	0.028 (5)	0.041 (6)	−0.012 (5)	−0.017 (5)	0.001 (4)
C1D	0.026 (5)	0.040 (6)	0.055 (7)	−0.002 (5)	−0.014 (5)	−0.009 (5)
C6D	0.086 (11)	0.021 (5)	0.052 (8)	−0.012 (6)	−0.037 (8)	0.005 (5)
C20B	0.209 (18)	0.122 (11)	0.044 (10)	−0.031 (11)	−0.027 (11)	0.030 (8)
C21B	0.167 (10)	0.097 (9)	0.124 (12)	−0.018 (7)	−0.067 (9)	0.017 (7)
C20D	0.16 (2)	0.113 (19)	0.023 (12)	−0.050 (15)	−0.022 (15)	0.026 (10)
C21D	0.18 (2)	0.19 (3)	0.077 (17)	−0.01 (2)	−0.029 (15)	−0.006 (19)
C20A	0.13 (2)	0.095 (19)	0.096 (18)	0.045 (14)	−0.056 (13)	−0.068 (15)
C21A	0.097 (19)	0.20 (5)	0.075 (15)	−0.010 (16)	−0.015 (13)	−0.06 (2)
C20C	0.100 (13)	0.122 (16)	0.093 (10)	−0.001 (9)	−0.036 (10)	−0.064 (8)
C21C	0.18 (3)	0.17 (2)	0.102 (11)	−0.035 (14)	−0.048 (16)	−0.025 (12)

### Geometric parameters (Å, º)

**Table:**

Br1—C10A	1.884 (4)	C5B—H5B1	0.9700
Br2—C10B	1.881 (4)	C5B—H5B2	0.9700
F1—C16A	1.349 (4)	C7B—C8B	1.360 (6)
F2—C16B	1.356 (4)	C7B—C12B	1.373 (6)
O1A—C2A	1.217 (4)	C7B—C6D	1.547 (13)
O2A—C19A	1.181 (5)	C7B—C6B	1.589 (11)
O3A—C19A	1.315 (5)	C8B—C9B	1.368 (6)
O3A—C20A	1.451 (10)	C8B—H8B	0.9300
O3A—C20C	1.469 (8)	C9B—C10B	1.366 (6)
C1A—C19A	1.512 (5)	C9B—H9B	0.9300
C1A—C2A	1.513 (5)	C10B—C11B	1.361 (6)
C1A—C6A	1.534 (5)	C11B—C12B	1.375 (6)
C1A—H1A	0.9800	C11B—H11B	0.9300
C2A—C3A	1.442 (5)	C12B—H12B	0.9300
C3A—C4A	1.344 (5)	C13B—C14B	1.392 (5)
C3A—H3A	0.9300	C13B—C18B	1.397 (5)
C4A—C13A	1.484 (5)	C14B—C15B	1.376 (5)
C4A—C5A	1.498 (5)	C14B—H14B	0.9300
C5A—C6A	1.523 (5)	C15B—C16B	1.366 (6)
C5A—H5A1	0.9700	C15B—H15B	0.9300
C5A—H5A2	0.9700	C16B—C17B	1.356 (6)
C6A—C7A	1.520 (5)	C17B—C18B	1.365 (5)
C6A—H6A	0.9800	C17B—H17B	0.9300
C7A—C8A	1.365 (5)	C18B—H18B	0.9300
C7A—C12A	1.375 (5)	C19B—C1B	1.544 (13)
C8A—C9A	1.379 (5)	C19B—C1D	1.548 (13)
C8A—H8A	0.9300	C1B—C6B	1.213 (16)
C9A—C10A	1.370 (6)	C1B—H1B	0.9800
C9A—H9A	0.9300	C6B—H6B	0.9800
C10A—C11A	1.355 (6)	C1D—C6D	1.350 (15)
C11A—C12A	1.377 (5)	C1D—H1D	0.9800
C11A—H11A	0.9300	C6D—H6D	0.9800
C12A—H12A	0.9300	C20B—C21B	1.502 (10)
C13A—C18A	1.380 (5)	C20B—H20C	0.9700
C13A—C14A	1.389 (5)	C20B—H20D	0.9700
C14A—C15A	1.375 (5)	C21B—H21A	0.9600
C14A—H14A	0.9300	C21B—H21B	0.9600
C15A—C16A	1.372 (6)	C21B—H21C	0.9600
C15A—H15A	0.9300	C20D—C21D	1.514 (10)
C16A—C17A	1.356 (6)	C20D—H20E	0.9700
C17A—C18A	1.373 (5)	C20D—H20F	0.9700
C17A—H17A	0.9300	C21D—H21D	0.9600
C18A—H18A	0.9300	C21D—H21E	0.9600
O1B—C2B	1.216 (4)	C21D—H21F	0.9600
O2B—C19B	1.176 (5)	C20A—C21A	1.503 (10)
O3B—C19B	1.306 (6)	C20A—H20A	0.9700
O3B—C20D	1.437 (10)	C20A—H20B	0.9700
O3B—C20B	1.449 (8)	C21A—H21G	0.9600
C2B—C3B	1.442 (5)	C21A—H21H	0.9600
C2B—C1B	1.546 (14)	C21A—H21I	0.9600
C2B—C1D	1.571 (13)	C20C—C21C	1.495 (10)
C3B—C4B	1.338 (5)	C20C—H20G	0.9700
C3B—H3B	0.9300	C20C—H20H	0.9700
C4B—C13B	1.476 (5)	C21C—H21J	0.9600
C4B—C5B	1.503 (5)	C21C—H21K	0.9600
C5B—C6D	1.482 (13)	C21C—H21L	0.9600
C5B—C6B	1.574 (12)		
			
C19A—O3A—C20A	106.5 (12)	C9B—C10B—Br2	119.2 (4)
C19A—O3A—C20C	123.6 (11)	C10B—C11B—C12B	119.6 (4)
C19A—C1A—C2A	110.1 (3)	C10B—C11B—H11B	120.2
C19A—C1A—C6A	112.4 (3)	C12B—C11B—H11B	120.2
C2A—C1A—C6A	111.5 (3)	C7B—C12B—C11B	121.2 (4)
C19A—C1A—H1A	107.5	C7B—C12B—H12B	119.4
C2A—C1A—H1A	107.5	C11B—C12B—H12B	119.4
C6A—C1A—H1A	107.5	C14B—C13B—C18B	117.5 (3)
O1A—C2A—C3A	122.2 (4)	C14B—C13B—C4B	122.2 (3)
O1A—C2A—C1A	120.0 (4)	C18B—C13B—C4B	120.4 (3)
C3A—C2A—C1A	117.8 (3)	C15B—C14B—C13B	121.1 (4)
C4A—C3A—C2A	123.9 (4)	C15B—C14B—H14B	119.5
C4A—C3A—H3A	118.1	C13B—C14B—H14B	119.5
C2A—C3A—H3A	118.1	C16B—C15B—C14B	118.7 (4)
C3A—C4A—C13A	121.0 (3)	C16B—C15B—H15B	120.7
C3A—C4A—C5A	120.3 (3)	C14B—C15B—H15B	120.7
C13A—C4A—C5A	118.7 (3)	C17B—C16B—F2	118.6 (4)
C4A—C5A—C6A	113.4 (3)	C17B—C16B—C15B	122.4 (4)
C4A—C5A—H5A1	108.9	F2—C16B—C15B	119.0 (4)
C6A—C5A—H5A1	108.9	C16B—C17B—C18B	118.9 (4)
C4A—C5A—H5A2	108.9	C16B—C17B—H17B	120.6
C6A—C5A—H5A2	108.9	C18B—C17B—H17B	120.6
H5A1—C5A—H5A2	107.7	C17B—C18B—C13B	121.5 (4)
C7A—C6A—C5A	111.7 (3)	C17B—C18B—H18B	119.2
C7A—C6A—C1A	113.2 (3)	C13B—C18B—H18B	119.2
C5A—C6A—C1A	110.7 (3)	O2B—C19B—O3B	124.2 (4)
C7A—C6A—H6A	107.0	O2B—C19B—C1B	138.0 (10)
C5A—C6A—H6A	107.0	O3B—C19B—C1B	97.8 (9)
C1A—C6A—H6A	107.0	O2B—C19B—C1D	109.0 (7)
C8A—C7A—C12A	116.4 (4)	O3B—C19B—C1D	126.7 (7)
C8A—C7A—C6A	120.3 (4)	C6B—C1B—C19B	127.8 (9)
C12A—C7A—C6A	123.3 (4)	C6B—C1B—C2B	122.4 (9)
C7A—C8A—C9A	122.1 (4)	C19B—C1B—C2B	106.2 (10)
C7A—C8A—H8A	119.0	C6B—C1B—H1B	96.3
C9A—C8A—H8A	119.0	C19B—C1B—H1B	96.3
C10A—C9A—C8A	120.0 (4)	C2B—C1B—H1B	96.3
C10A—C9A—H9A	120.0	C1B—C6B—C5B	123.0 (9)
C8A—C9A—H9A	120.0	C1B—C6B—C7B	121.9 (9)
C11A—C10A—C9A	119.2 (4)	C5B—C6B—C7B	105.1 (8)
C11A—C10A—Br1	121.4 (4)	C1B—C6B—H6B	100.5
C9A—C10A—Br1	119.4 (3)	C5B—C6B—H6B	100.5
C10A—C11A—C12A	119.8 (4)	C7B—C6B—H6B	100.5
C10A—C11A—H11A	120.1	C6D—C1D—C19B	124.6 (10)
C12A—C11A—H11A	120.1	C6D—C1D—C2B	117.0 (9)
C7A—C12A—C11A	122.4 (4)	C19B—C1D—C2B	104.7 (9)
C7A—C12A—H12A	118.8	C6D—C1D—H1D	102.4
C11A—C12A—H12A	118.8	C19B—C1D—H1D	102.4
C18A—C13A—C14A	117.5 (3)	C2B—C1D—H1D	102.4
C18A—C13A—C4A	121.3 (4)	C1D—C6D—C5B	124.0 (9)
C14A—C13A—C4A	121.1 (3)	C1D—C6D—C7B	119.6 (9)
C15A—C14A—C13A	121.3 (4)	C5B—C6D—C7B	112.0 (10)
C15A—C14A—H14A	119.4	C1D—C6D—H6D	97.0
C13A—C14A—H14A	119.4	C5B—C6D—H6D	97.0
C16A—C15A—C14A	118.4 (4)	C7B—C6D—H6D	97.0
C16A—C15A—H15A	120.8	O3B—C20B—C21B	105.3 (9)
C14A—C15A—H15A	120.8	O3B—C20B—H20C	110.7
F1—C16A—C17A	118.5 (4)	C21B—C20B—H20C	110.7
F1—C16A—C15A	119.1 (4)	O3B—C20B—H20D	110.7
C17A—C16A—C15A	122.4 (4)	C21B—C20B—H20D	110.7
C16A—C17A—C18A	118.3 (4)	H20C—C20B—H20D	108.8
C16A—C17A—H17A	120.9	C20B—C21B—H21A	109.5
C18A—C17A—H17A	120.9	C20B—C21B—H21B	109.5
C17A—C18A—C13A	122.1 (4)	H21A—C21B—H21B	109.5
C17A—C18A—H18A	119.0	C20B—C21B—H21C	109.5
C13A—C18A—H18A	119.0	H21A—C21B—H21C	109.5
O2A—C19A—O3A	124.3 (4)	H21B—C21B—H21C	109.5
O2A—C19A—C1A	124.7 (5)	O3B—C20D—C21D	109.3 (18)
O3A—C19A—C1A	111.0 (4)	O3B—C20D—H20E	109.8
C19B—O3B—C20D	101.7 (9)	C21D—C20D—H20E	109.8
C19B—O3B—C20B	124.3 (10)	O3B—C20D—H20F	109.8
O1B—C2B—C3B	122.9 (4)	C21D—C20D—H20F	109.8
O1B—C2B—C1B	120.9 (5)	H20E—C20D—H20F	108.3
C3B—C2B—C1B	114.3 (5)	C20D—C21D—H21D	109.5
O1B—C2B—C1D	120.2 (5)	C20D—C21D—H21E	109.5
C3B—C2B—C1D	114.8 (5)	H21D—C21D—H21E	109.5
C4B—C3B—C2B	123.8 (3)	C20D—C21D—H21F	109.5
C4B—C3B—H3B	118.1	H21D—C21D—H21F	109.5
C2B—C3B—H3B	118.1	H21E—C21D—H21F	109.5
C3B—C4B—C13B	121.1 (3)	O3A—C20A—C21A	105 (2)
C3B—C4B—C5B	120.6 (3)	O3A—C20A—H20A	110.6
C13B—C4B—C5B	118.3 (3)	C21A—C20A—H20A	110.6
C6D—C5B—C4B	115.2 (5)	O3A—C20A—H20B	110.6
C4B—C5B—C6B	111.5 (5)	C21A—C20A—H20B	110.6
C6D—C5B—H5B1	82.3	H20A—C20A—H20B	108.8
C4B—C5B—H5B1	109.3	C20A—C21A—H21G	109.5
C6B—C5B—H5B1	109.3	C20A—C21A—H21H	109.5
C6D—C5B—H5B2	127.5	H21G—C21A—H21H	109.5
C4B—C5B—H5B2	109.3	C20A—C21A—H21I	109.5
C6B—C5B—H5B2	109.3	H21G—C21A—H21I	109.5
H5B1—C5B—H5B2	108.0	H21H—C21A—H21I	109.5
C8B—C7B—C12B	117.9 (4)	O3A—C20C—C21C	101.9 (15)
C8B—C7B—C6D	135.5 (9)	O3A—C20C—H20G	111.4
C12B—C7B—C6D	106.4 (9)	C21C—C20C—H20G	111.4
C8B—C7B—C6B	109.8 (7)	O3A—C20C—H20H	111.4
C12B—C7B—C6B	132.1 (7)	C21C—C20C—H20H	111.4
C7B—C8B—C9B	121.8 (4)	H20G—C20C—H20H	109.3
C7B—C8B—H8B	119.1	C20C—C21C—H21J	109.5
C9B—C8B—H8B	119.1	C20C—C21C—H21K	109.5
C10B—C9B—C8B	119.5 (4)	H21J—C21C—H21K	109.5
C10B—C9B—H9B	120.2	C20C—C21C—H21L	109.5
C8B—C9B—H9B	120.2	H21J—C21C—H21L	109.5
C11B—C10B—C9B	120.0 (4)	H21K—C21C—H21L	109.5
C11B—C10B—Br2	120.9 (4)		
			
C19A—C1A—C2A—O1A	25.6 (6)	C18B—C13B—C14B—C15B	1.3 (6)
C6A—C1A—C2A—O1A	151.1 (4)	C4B—C13B—C14B—C15B	179.7 (4)
C19A—C1A—C2A—C3A	−156.1 (4)	C13B—C14B—C15B—C16B	−0.1 (7)
C6A—C1A—C2A—C3A	−30.5 (5)	C14B—C15B—C16B—C17B	−1.5 (8)
O1A—C2A—C3A—C4A	−176.8 (4)	C14B—C15B—C16B—F2	180.0 (4)
C1A—C2A—C3A—C4A	4.9 (6)	F2—C16B—C17B—C18B	−179.8 (4)
C2A—C3A—C4A—C13A	177.2 (3)	C15B—C16B—C17B—C18B	1.7 (8)
C2A—C3A—C4A—C5A	−1.6 (6)	C16B—C17B—C18B—C13B	−0.3 (7)
C3A—C4A—C5A—C6A	24.7 (5)	C14B—C13B—C18B—C17B	−1.1 (6)
C13A—C4A—C5A—C6A	−154.1 (3)	C4B—C13B—C18B—C17B	−179.5 (4)
C4A—C5A—C6A—C7A	−176.5 (3)	C20D—O3B—C19B—O2B	7.7 (17)
C4A—C5A—C6A—C1A	−49.5 (4)	C20B—O3B—C19B—O2B	−2.4 (10)
C19A—C1A—C6A—C7A	−57.6 (4)	C20D—O3B—C19B—C1B	−171.8 (17)
C2A—C1A—C6A—C7A	178.2 (3)	C20B—O3B—C19B—C1B	178.1 (9)
C19A—C1A—C6A—C5A	176.2 (3)	C20D—O3B—C19B—C1D	−168.8 (17)
C2A—C1A—C6A—C5A	52.0 (4)	C20B—O3B—C19B—C1D	−178.9 (9)
C5A—C6A—C7A—C8A	−93.4 (4)	O2B—C19B—C1B—C6B	82 (2)
C1A—C6A—C7A—C8A	141.0 (4)	O3B—C19B—C1B—C6B	−98.4 (16)
C5A—C6A—C7A—C12A	85.6 (5)	C1D—C19B—C1B—C6B	86.5 (17)
C1A—C6A—C7A—C12A	−40.0 (5)	O2B—C19B—C1B—C2B	−76.4 (12)
C12A—C7A—C8A—C9A	0.3 (6)	O3B—C19B—C1B—C2B	103.0 (11)
C6A—C7A—C8A—C9A	179.4 (4)	C1D—C19B—C1B—C2B	−72 (2)
C7A—C8A—C9A—C10A	2.1 (7)	O1B—C2B—C1B—C6B	173.6 (10)
C8A—C9A—C10A—C11A	−3.2 (7)	C3B—C2B—C1B—C6B	8.5 (18)
C8A—C9A—C10A—Br1	177.6 (3)	C1D—C2B—C1B—C6B	−89.1 (16)
C9A—C10A—C11A—C12A	1.8 (7)	O1B—C2B—C1B—C19B	−26.3 (16)
Br1—C10A—C11A—C12A	−178.9 (4)	C3B—C2B—C1B—C19B	168.5 (8)
C8A—C7A—C12A—C11A	−1.7 (7)	C1D—C2B—C1B—C19B	71 (2)
C6A—C7A—C12A—C11A	179.2 (4)	C19B—C1B—C6B—C5B	−146.3 (18)
C10A—C11A—C12A—C7A	0.7 (7)	C2B—C1B—C6B—C5B	9.1 (18)
C3A—C4A—C13A—C18A	−24.5 (5)	C19B—C1B—C6B—C7B	−6 (2)
C5A—C4A—C13A—C18A	154.3 (4)	C2B—C1B—C6B—C7B	149.5 (16)
C3A—C4A—C13A—C14A	158.6 (4)	C6D—C5B—C6B—C1B	81.1 (17)
C5A—C4A—C13A—C14A	−22.6 (5)	C4B—C5B—C6B—C1B	−22.5 (12)
C18A—C13A—C14A—C15A	0.6 (6)	C6D—C5B—C6B—C7B	−64.7 (15)
C4A—C13A—C14A—C15A	177.6 (4)	C4B—C5B—C6B—C7B	−168.4 (5)
C13A—C14A—C15A—C16A	−0.4 (7)	C8B—C7B—C6B—C1B	114.5 (11)
C14A—C15A—C16A—F1	−178.3 (4)	C12B—C7B—C6B—C1B	−59.3 (13)
C14A—C15A—C16A—C17A	0.3 (7)	C6D—C7B—C6B—C1B	−84.3 (18)
F1—C16A—C17A—C18A	178.1 (4)	C8B—C7B—C6B—C5B	−99.1 (8)
C15A—C16A—C17A—C18A	−0.5 (7)	C12B—C7B—C6B—C5B	87.0 (8)
C16A—C17A—C18A—C13A	0.8 (6)	C6D—C7B—C6B—C5B	62.0 (15)
C14A—C13A—C18A—C17A	−0.8 (6)	O2B—C19B—C1D—C6D	106.5 (10)
C4A—C13A—C18A—C17A	−177.8 (4)	O3B—C19B—C1D—C6D	−76.5 (12)
C20A—O3A—C19A—O2A	−8 (2)	C1B—C19B—C1D—C6D	−70.4 (15)
C20C—O3A—C19A—O2A	7.8 (12)	O2B—C19B—C1D—C2B	−114.7 (7)
C20A—O3A—C19A—C1A	173.0 (19)	O3B—C19B—C1D—C2B	62.3 (9)
C20C—O3A—C19A—C1A	−171.1 (10)	C1B—C19B—C1D—C2B	68.4 (16)
C2A—C1A—C19A—O2A	−110.4 (5)	O1B—C2B—C1D—C6D	173.3 (9)
C6A—C1A—C19A—O2A	124.6 (5)	C3B—C2B—C1D—C6D	−22.7 (11)
C2A—C1A—C19A—O3A	68.6 (4)	C1B—C2B—C1D—C6D	73.2 (15)
C6A—C1A—C19A—O3A	−56.4 (5)	O1B—C2B—C1D—C19B	30.8 (10)
O1B—C2B—C3B—C4B	−176.6 (4)	C3B—C2B—C1D—C19B	−165.3 (5)
C1B—C2B—C3B—C4B	−11.8 (11)	C1B—C2B—C1D—C19B	−69.4 (16)
C1D—C2B—C3B—C4B	19.9 (8)	C19B—C1D—C6D—C5B	143.6 (17)
C2B—C3B—C4B—C13B	176.6 (3)	C2B—C1D—C6D—C5B	9.3 (18)
C2B—C3B—C4B—C5B	−2.9 (6)	C19B—C1D—C6D—C7B	−10.9 (17)
C3B—C4B—C5B—C6D	−11.8 (11)	C2B—C1D—C6D—C7B	−145.3 (15)
C13B—C4B—C5B—C6D	168.8 (10)	C4B—C5B—C6D—C1D	7.8 (18)
C3B—C4B—C5B—C6B	18.8 (7)	C6B—C5B—C6D—C1D	−81.0 (16)
C13B—C4B—C5B—C6B	−160.7 (6)	C4B—C5B—C6D—C7B	164.0 (8)
C12B—C7B—C8B—C9B	−0.4 (6)	C6B—C5B—C6D—C7B	75 (2)
C6D—C7B—C8B—C9B	172.3 (7)	C8B—C7B—C6D—C1D	105.7 (13)
C6B—C7B—C8B—C9B	−175.3 (5)	C12B—C7B—C6D—C1D	−81.0 (13)
C7B—C8B—C9B—C10B	0.9 (7)	C6B—C7B—C6D—C1D	79.9 (14)
C8B—C9B—C10B—C11B	−0.7 (6)	C8B—C7B—C6D—C5B	−51.8 (16)
C8B—C9B—C10B—Br2	178.9 (3)	C12B—C7B—C6D—C5B	121.6 (12)
C9B—C10B—C11B—C12B	0.1 (7)	C6B—C7B—C6D—C5B	−78 (2)
Br2—C10B—C11B—C12B	−179.6 (3)	C19B—O3B—C20B—C21B	98.4 (16)
C8B—C7B—C12B—C11B	−0.3 (7)	C20D—O3B—C20B—C21B	74 (4)
C6D—C7B—C12B—C11B	−175.0 (5)	C19B—O3B—C20D—C21D	−179 (3)
C6B—C7B—C12B—C11B	173.2 (7)	C20B—O3B—C20D—C21D	−20 (3)
C10B—C11B—C12B—C7B	0.5 (7)	C19A—O3A—C20A—C21A	−161 (4)
C3B—C4B—C13B—C14B	156.9 (4)	C20C—O3A—C20A—C21A	56 (5)
C5B—C4B—C13B—C14B	−23.6 (5)	C19A—O3A—C20C—C21C	−99 (2)
C3B—C4B—C13B—C18B	−24.7 (5)	C20A—O3A—C20C—C21C	−55 (5)
C5B—C4B—C13B—C18B	154.7 (3)		

### Hydrogen-bond geometry (Å, º)

**Table:**

*D*—H···*A*	*D*—H	H···*A*	*D*···*A*	*D*—H···*A*
C5*A*—H5*A*1···O1*B*^i^	0.97	2.58	3.388 (5)	141
C14*A*—H14*A*···O1*B*^i^	0.93	2.58	3.445 (6)	154
C5*B*—H5*B*1···O1*A*^ii^	0.97	2.55	3.351 (4)	140
C5*B*—H5*B*2···O2*A*^iii^	0.97	2.59	3.457 (5)	149

Symmetry codes: (i) *x*, *y*−1, *z*−1; (ii) *x*, *y*, *z*+1; (iii) −*x*+2, −*y*+1, −*z*+1.
